# A Machine Learning and Deep Learning Approach for Recognizing Handwritten Digits

**DOI:** 10.1155/2022/9869948

**Published:** 2022-07-15

**Authors:** Ayushi Sharma, Harshit Bhardwaj, Arpit Bhardwaj, Aditi Sakalle, Divya Acharya, Wubshet Ibrahim

**Affiliations:** ^1^Department of Computer Science and Engineering SoE, Galgotias University, Greater Noida, India; ^2^Department of Computer Science and Engineering, BML Munjal University, Gurugram, India; ^3^Department of Computer Science and Engineering, Gautam Buddha University, Greater Noida, India; ^4^HCL Technology Limited, Noida, India; ^5^Department of Mathematics, Ambo University, Ambo, Ethiopia

## Abstract

Optical character recognition (OCR) can be a subcategory of graphic design that involves extracting text from images or scanned documents. We have chosen to make unique handwritten digits available on the Modified National Institute of Standards and Technology website for this project. The Machine Learning and Depp Learning algorithms are used in this project to measure the accuracy of handwritten displays of letters and numbers. Also, we show the classification accuracy comparison between them. The results showed that the CNN classifier achieved the highest classification accuracy of 98.83%.

## 1. Introduction

It is straightforward for the human brain to reuse images and examine them. Whenever the human brain sees anything, it recognizes its moments and whenever anything like that regenerates memory and recollection. This is because of the human brain's analytical behavior, which also compares memories with new ones. An efficient robot and this technology are called image processing (a way of absorbing images and other types of digital code that can be reused by a computer using a specific algorithm that improves the quality of that quickly recovered memory/image) [1]. Image processing is used in many places to identify and correct people. Image processing is a field related to image analysis for more helpful information from them. This method takes images into computer-readable forms, uses specific algorithms, and keeps them in the best images or with several features that may be accustomed to rewarding valuable information. Image processing is used in several areas, especially at present, and several other software developments use this concept. For example, we now have toned buses that will critique other buses and mortal creatures to avoid accidents. Also, some social media platforms, such as Facebook, can create face recognition because of this fashion [2].

Similarly, some software uses it to honor characters in other images, namely the optic character recognition concept, which we will explore and experience during this design. One of the narrow image processing fields downloads the letters to the image, which is matched to Optical Character Recognition (OCR) [3]. This process involves reading a picture containing one or more characters or reading a well-researched or handwritten book worth handling. Many experiments have become obsolete in this field to investigate the proper methods for maximum enjoyment and fairness. Standard algorithms have proven high performance in machine-readable algorithms such as Networks Neural and Vector Support Machine. The logic behind the support vector machine algorithm is to detect a hyperplane in the N-dimensional space (N—number of elements) that clearly distinguishes data points. Hyperplanes are decision parameters that help to separate data points. Data points that fall on both sides can be defined in different classes.

Similarly, the size of a hyperplane depends on several factors. If the input value is 2, the hyperplane is just a line. When the input value is 3, the hyperplane becomes a two-dimensional plane. It is hard to imagine if the number of items exceeds 3. KNN is one of the easiest algorithms to learn. The nearest neighbor is not a parameter. It makes no assumptions about the original data. The KNN is also called lazy algorithms because they are not learned in the training phase but retain data points but are learned in the testing phase. This is a distance-based algorithm [4]. Decision tree algorithms are in the field of supervised learning. It can be used to solve retrieval as well as editing problems. Decision trees use a tree view to solve a problem where each leaf area corresponds to a class label, and the structures are displayed inside the tree. Random forest is an integrated system that can perform retrofitting and split operations using multiple cutting trees and a method called Bootstrap and Aggregation, commonly known as bagging [5]. The basic premise is to combine numerous decision trees to determine the result rather than rely on each decision tree. One of the many OCR functions is to download handwritten characters. We will direct you to set up media that will handle handwritten numbers during this project. We will be reading pictures that contain handwritten numbers from the MNIST website and will try to say what number that picture represents. We will use the presentation methods of Image Matching, which are also associated with Matrix Matching, SVM, KNN, decision tree, random forest, Gaussian Naïve Bayes, Genetic Programming, and Convolutional Neural Network. This project aims to use and manipulate the techniques of the presentation image to build the system and continue to Polish and improve it to investigate how much it can be improved.

## 2. Literature Survey

A great deal of work has been achieved in handwriting popularity, thinking about many extraordinary languages, and using alternative techniques and algorithms. For example, Arora et al. [6] feature names, numerical boundaries, that is, boundary, completed area, axial length, axial length, contact continuity, and Levenberg–Marquard resonance training as opposed to training neural networks—algorithms used. They obtained a high sensitivity of 93,130 samples using 50 subclasses of retired neurons.

Jannoud [7] Euler's numerical calculations are considered. A more sophisticated birth point model is suggested that can provide better-crafted improvements with less training and support time than the original model. The oblique-based birth point model has a technology of 98.5. In this case, the loading matrix is trained using the neural network's write-read rules, which is why visual recognition is calculated. The system has a high degree of cunning, but exceptions will be accepted for the same characters. Therefore, the Euclidean space condition determines the type of characters that can be compared.

In Bishop [8], I have tried the striking construction styles and found that diagonal, oblique, and direction are the most accurate, and some are combined to make small-scale improvements. The perceptron literacy software utilized by Kumar Patel et al. [9] is Character recognition. The line dividing line was used for the point of birth, and 80 was obtained. The Euclidean range was used for character recognition, and 90-degree sensitivity was obtained using ocean morphometric multiresolution. Some samples are correctly separated by 100 fineness, while others are separated by complete relative error.

Kermorvant et al. [10] introduced an entirely new way of recognizing handwritten and offline character characters using a noncontinuous neural network. They compared the results with the use of a locally based HMM system. Tests show that the new method works better with a basic HMM system and is more robust in changing the sample size.

Deshpande et al. [11] used the systems proposed by the brackets of other Devanagari character brackets and have released the rules for series chart items and integrated objects. The present-day accuracy located in the collection code separator is 88.19%, and the essential overflow section is also 65.67%. At the same time, the combination of double splits and the usage of a massive quantity of big charts lets in 98.03%accuracy. Twelve special categories and four units of capabilities (two sets of grey binary images) are recognized by Devanagari characters. The results display a reflection (millet) quality of 12 sections with an average accuracy of 94.94%. In addition, they concluded that bending features produce special effects ingredient features for nearly all magnificence dividers.

Gajjar et al. [12] use the Devanagari characters and started to be diagnosed through the simulation of commonplace speech. If they do not feel healthy in any sample, they're transferred to a small distance, editing precise out, so the overall accuracy is 82%. The SVM and ANN separators were used for code graphs, a series were based on shadow, very long, and the Devanagari character visibility has market-based features. They found that reliable separation could be achieved through SVM.

Rani and Singh [13] get the best 99.4% accuracy using different component set combinations. Two separate components were set as compounds of Diagonal and background distribution function and Histogram display, diagonal function, and a back-to-back distribution.

Pranob et al. [14] used the Antminer algorithm to locate Thai characters, and 97% of the training set is recognizable. In this study, a comparative work of Gabor's work related to gradient work was performed to identify Chinese manuscripts and fully realized that gradient work was much better than Gradient work.

Ashlin Deepa et al. [15] proposed a handwritten Arabic manuscript system that used the Discrete Wavelet Transform to extract the feature and thus the best recognition for single characters (99%). However, the recognition rate was worse (91%) for central characters.

Deshmukh and Ragha [16] discuss the order of the graph-by-bottom approach to extracting letters from printed Devanagari texts. First, removing the characters commonly used as binary or other advanced features will be computerized to differentiate the trainer. The author also discusses a few Euler, symmetrical, and class divisions structures at each level. This method works for families with unchanged fonts. Next, the authors proposed a UNHCR program with a coefficient of integration. Although it brings good results, it is widely calculated for consistency. In addition, a sequential code approach has been proposed in the document, in which the accuracy of serial code function is tested by neurological retro enhancing networks and supporting vectors.

Pradeep et al.'s [17] use of three different types of 4044, namely 4044 Density object, Pulse object, and 4044 Component Descriptor object, have been used to separate the Devanagari data. The composite structure of multiple class dividers is proposed to increase the recognition's reliability and obtain 89.6% accuracy in Devanagari handwritten numbers. Sandhya Arora used four techniques to remove the features, namely crossroads.

## 3. Methodology

There are several classifiers that we used which are discussed below.

### 3.1. Dataset

**Figure 1:**
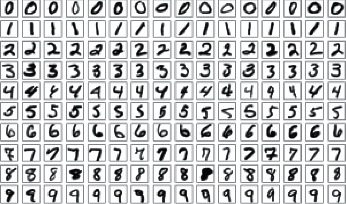
Standard MNIST dataset.

The Standard MNIST database [18], published by Yann LeCun of the Courant Institute at New York University, is the central database used to train separators. A training set with 60,000 labels and a test set of 10,000 labels are included in the database. Handwritten data samples come from about 250 different authors, and the test set takes samples of entirely different authors. There is no conflict between the test set and the authors of the training set. The MNIST dataset shown in Figure 1, is rendered as binary files in the IDX file format, which visually represents the numbers and contains significant representations of the dataset. Data were read in the image matrix. Each image was subject to a primary orientation method where the value of each pixel was divided by the maximum pixel value of the sample.

### 3.2. Support Vector Machine

A guide vector system was proposed using Vapnik as a dual classifier (CV95). It represents one of the most remote-monitored readability classifiers, and it is undoubtedly applicable across a wide range of operations. SVM discovered a hyperplane separating the recordings from the special lessons. Each case is evaluated by one of each lesson, and they are represented as factors inboxes. SVM builds an instance-based version of a field group, and another portion of unspecified instances is completed using this instance. The following equation describes the hyperplane separating the classified instances from the school set: xi Rd are examples represented as vectors in the d-dimensional region, *n* is just the range *d*′ instance, and *y*_*i*_ is the location of the respective instances. Examples and *w* and *b* are the parameters of the hyperplane. This hyperplane is prepared using the closest examples expressed as a guiding vector. The hyperplane should be as far as possible from the examples of all the lessons space to be maximized 2/||*W*||.

The defined version covers the problem of classifying authentic lifestyle profiles because all the examples must be exactly closer to the hyperplane and includes global records of noise and often exceptions. Is the version not suitable for separating comparable records? Therefore, the use of soggy perimeters is suggested for this complexity. External soft limits are hired instead. The idea is to introduce a slack variable that allows some examples to be misclassified, that is, not exactly closer to the hyperplane. It is described using the following expression. This hyperplane termination is terminated using the next quadratic programming problem, where *c* is the smooth edge parameter. Adding a pretty good value of the *c* parameter without any signs or symptoms moves the instance to harsh environments. The selection of the associated costs for this parameter includes the primary influence on the level of support sophistication [19].

### 3.3. Decision Tree

The root of the selection tree is shown the wrong way up. The bold textual content in black represents the placement/node inside the photograph on the left, based totally on wherein the tree splits into branches/edges. The selection/leaf, in this situation, whether the passenger is lifeless or alive, is displayed as red and green text, respectively, on the cease of the indistinguishable branch. Although an accurate dataset will have many attributes, and this will be a branch in a huge tree, the simplicity of this process cannot be ignored. The value of each element is clearly stated, and the relationship can be easily viewed [20].

### 3.4. Random Forest

Random Forest is a well-known machine-learning algorithm that uses supervised learning techniques. It can be used for both editing and retrieval problems in machine learning. It is based on integrated learning, which is a way to incorporate multiple dividers to solve a complex issue and maximize model performance. “Random Forest is a subdivision that contains the number of decision trees in the various datasets provided and takes measures to improve the predicted accuracy of that data,” according to the term. Instead of relying on a single decision tree, the random forest collects predictions from each tree and predicts the outcome based on multiple predictable votes [21].

### 3.5. K-Nearest Neighbour

K-nearest neighbor is a Supervised gadget mastering set of rules that's one of the maximum basic. The KNN rules assume that the new case/information and present situations are equal and place the brand-new case within the same category as the existing classes. The KNN approach stores all available information and separates a new information point based on its similarity to current data. This means new facts may be speedy looked after in the correct class using the KNN approach. The KNN rules can be used for retransmission and partition; however, they are regularly used for partition operations. The KNN algorithm is a nonparametric set of rules; this means that it does now not reflect on consideration of the information. It is also referred to as a lazy scholar algorithm as it does now not learn from the training right away set; as an alternative, it saves the database and takes motion on it all through the break up [24].

### 3.6. Gaussian Naive Bayes

The Naive Bayes Approach is a supervised learning approach to problem-solving based on a Bayes perspective. It is widely used to classify text into large training databases. Naive Bayes Classifier is an easy and powerful method of the class that facilitates the development of fast device mastering models that could speedy wager. It classifies feasible classes, making predictions based totally on the probability of an item. Spam filtering, mood analysis, and name editing are examples of the Naive Bayes rules [22].

### 3.7. Genetic Programming

GP is a form of Evolutionary Algorithm (EA). EAs are employed to find immediate solutions to problems that people are unable to address. The adaptive nature of EAs can yield solutions that are comparable to, and often better than, human efforts since they are free of human prejudices or biases. GP software systems use a random mutation, crossover, a fitness function, and numerous generations of evolution to perform a user-defined job, which is inspired by biological evolution and its core mechanics [23–25].

### 3.8. Convolutional Neural Network (CNN)

A convolutional neural network (CNN or ConvNet) is a community architecture for deep studying which learns without delay from information, removing the want for manual function extraction. CNNs are beneficial for finding patterns in snapshots to recognize objects, faces, and scenes. They can also be quite powerful for classifying nonimage statistics, including audio, time collection, and sign information. In addition, packages that call for item recognition and pc vision—such as self-riding automobiles and face-recognition packages—depend heavily on CNN [26–28].

## 4. Experimental Results

A few scanned sites from the National Institute of Standards and Technology are being used to create a database (NIST). The website is known as Modified NIST or MNIST dataset. Digital images are exported to various scanned, resized, and embedded pages. This makes it an excellent website for test models because it calls for minimal fact cleaning or refining, which lets the engineer to conscious of machine studying. Every photo is a square of 28 pixels through 28 pixels (784 pixels overall). A standard website tests and examines models, including 60,000 images used for model training and the second set of 10,000 images used for testing. It is a digital recognition function. As a result, ten numbers (0–9) need to be predicted, or 10 categories. Predictability error, which does not exceed the accuracy of distorted sections, is used to report results.

**Figure 2:**
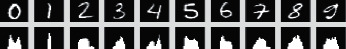
Projection of histogram on the *x*-axis (*y* = 0).

In this, we see the use of the notability chart as a set of levels for handwritten metrics. Featured graphs are commonly used to prevalence classified integers (similar to registration code identification). However, identity prominence graphs have not been widely used for handwritten integers, especially those with no other feature set. We intentionally used this weak feature set to verify our classifiers under adverse conditions.  Figure 2 illustrates the histogram featured on the *x*-axis for all 10 points.

**Figure 3:**
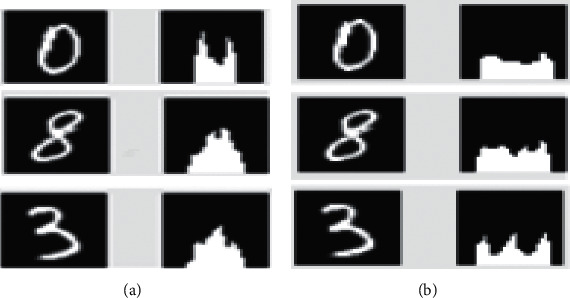
Diagram illustrates figures 0, 8, and 3 on (a) *x* and (b) *y* axes.

Due to different notation styles, pen consistency, angles, etc., the featured graphs may differ for the same number. Otherwise, they will be similar to other raw materials, so the overhang on one shaft may not be sufficient.  Figure 3 illustrates the histogram for points 0, 3, and 8.

**Figure 4:**
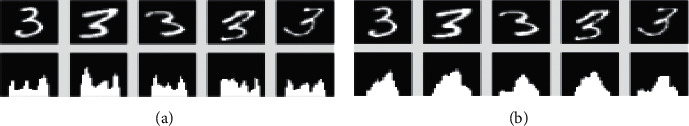
Graph of digit 3 on (a) *x* axis (b) *y* axis.

**Figure 5:**
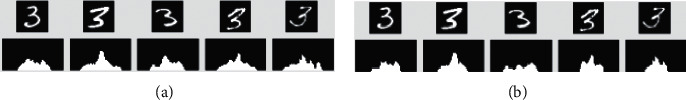
Diagram of number 3 on (a) *y* = *x* and (b) *y* = −*x*.

In the Degree histogram, the *X*-axis highlight for points 8 and 3 is similar, with the vertex in the middle, but the *Y*-axis highlight is different. Number 3 has three peaks, while number 8 incorporates a slight bump in the middle. The prominence histogram on opposite points 8 and 0 on the *y*-axis is the same, but the difference is evident in the prominence plot on the *x*-axis. In addition to these two featured graphs, in our algorithm, we have used two other featured graphs, on the lines *y* = *x* and *y* = −*x*, thus, representing each number by four histograms. Figures 4 and 5 show examples of four histograms for different samples of the digit 3. The histograms featured on one axis can vary greatly but have identical characteristics. Therefore, combining the four charts makes it possible to separate the different metrics. The described set of points is used as input to all the classifiers. Recognition of handwritten digits requires 10 distinct positions, with one preceding sign for each number. There are two main methods used in similar cases. The first, called one against all, builds a model for each class. Each sample separates a class from all other classes. This method is more suitable for classifiers that generate real values. However, a model for several classes, if there are *n* classes plus *n*(*n *−* *1)/2 and the model must be established. The division of the unknown archetype can be determined by counting the votes. Each model produces the result, and also, the most solved class in that time represents the unknown case type. Even when two or more classes have the same number of votes, different styles are used to decide. In this article, we have proposed a mixture of the two mentioned methods for multiclassification. Initially, classes were predicted by 10 different models (one against all). However, we used a unique method if the category is not uniquely identified or is not defined in any way. An essential part of the parenthesis process is expansion. The training and test data score values should be measured (+) or (−). The scale value has a significant impact on the subtlety of the brackets. Covering information in the lower range of numbers will dominate the information in the lower range. Training and test data should also be evaluated with the same factor.

**Figure 6:**
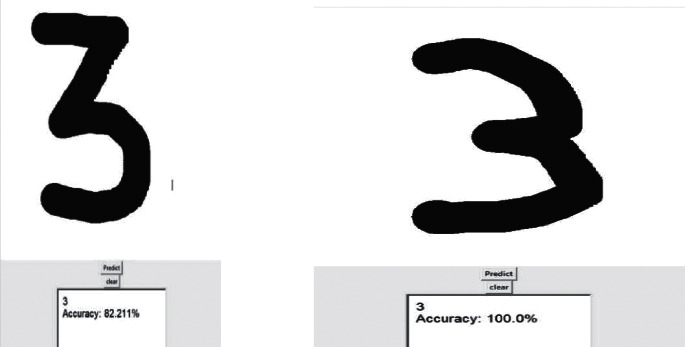
Change in accuracy due to change in the shape of the digit.

If you draw any of the numbers from your trackpad of the computer, the computer will predict the number itself and tell you the number it indicates with the accuracy number, and it differs by the shape you draw on your trackpad. The change in classification accuracy due to change in shape of the digit is shown in Figure 6.

**Figure 7:**
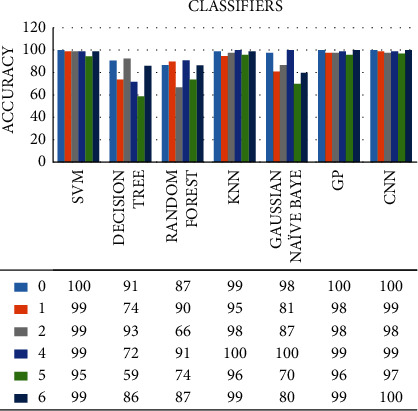
Comparison between different classifiers classification accuracy.

Here, we take a look at the accuracies of different classifiers shown in Figure 7. We take 1 to 6 digits and then analyze the accuracy of other numbers with various classifiers, where we found out that CNN has the most significant accuracy among all the classifiers. After CNN, SVM has the second-largest accuracy. CNN has the highest accuracy of 100% at digit 0 and the least at digit 5. Similarly, SVM stands out to be the second-largest accurate classifier with the highest accuracy (100%) at digit 0 and the least (95%) at digit 5. The GP classifier has the highest classification accuracy of 100% for 0 digit and least of 96% for digit 5. Similarly, as shown in the figure, the decision tree has the highest accuracy (93%) at digit 2 but is the least accurate classifier among all the other classifiers. The random forest has the highest accuracy (91%) at digit 4 and the least (55%) at digit 3. KNN is the second-highest accurate classifier among all, with the highest accuracy (100%) at digit 4 and least (89%) at digit 3. Similarly, Gaussian Naive Bayes has the highest accuracy (100%) at digit 4 and least (80%) at digit 6.  Table 1 represents the comparison of performance measures of all the implemented classifiers for recognition of handwritten digits.

## 5. Conclusion

In this paper, we applied machine learning and deep learning techniques to predict the handwritten digits. Popular algorithms such as KNN, SVM, RFC, DECISION TREE, GNB, GP, and CNN were tested to analyze the differences between them. We are using keras as the backend and tensorflow as the software library. The CNN classifier outperforms the other classifier with a classification accuracy of 98.83% for the recognition of handwritten digits.

## Figures and Tables

**Table 1:** Comparison of sensitivity, precision, and specificity of SVM, decision tree, random forest, KNN, Gaussian Naive Bayes, GP, and CNN classifiers.

Classifier	Sensitivity (%)	Precision (%)	Specificity (%)
SVM	98.16	98.64	98.96
Decision tree	78.24	79.36	80.42
Random forest	81.72	82.88	83.64
KNN	97.18	97.92	98.58
Gaussian Naive Bayes	85.74	86.36	86.80
GP	98.10	98.58	98.92
CNN	98.48	98.86	99.14

## Data Availability

The data are available from the corresponding author upon request.
